# The influence of parent–child attachment on school adjustment among the left-behind children of overseas Chinese: The chain mediating role of peer relationships and hometown identity

**DOI:** 10.3389/fpsyg.2022.1041805

**Published:** 2022-11-09

**Authors:** Huilan Zhang, Zihan Li, Xiaoqiu Yan, Chunkao Deng

**Affiliations:** Department of Education, College of Education, Wenzhou University, Wenzhou, China

**Keywords:** left-behind children, parent–child attachment, school adjustment, peer relationships, hometown identity

## Abstract

**Background:**

The left-behind children of overseas Chinese are a kind of vulnerable children in the eastern coastal areas of China. Previous studies have shown that there are problems in their school adjustment. This study explored the relationship between parent–child attachment and school adaptation among the left-behind children of overseas Chinese parents, as well as the chain mediating role of peer relationships and hometown identity.

**Methods:**

A total of 1,047 students in grades 3–8 from 5 schools in Zhejiang Province were selected by cluster sampling. The cross-sectional survey was compiled from the Inventory of Parent and Peer Attachment, the Adaptation subscale of the Adolescent Mental Health Quality Questionnaire–Chinese Version, the Hometown Identity Scale, and the Student Peer Relationship Scale. Structural equation modeling was used to test the conceptual model.

**Results:**

The results showed that the influence of parent–child attachment on school adjustment among the left-behind children of overseas Chinese was mediated by hometown identity. Moreover, this impact was also sequentially mediated by peer relationships and hometown identity.

**Conclusion:**

This study revealed peer relationships and hometown identity as underlying mechanism that explained the influence of parent–child attachment on school adjustment among left-behind children. It may provide empirical support for future interventions.

## Introduction

Left-behind children of overseas Chinese refer to those who are left behind in China after their parents leaving the country, due to economic or family reasons (Su et al., 2013). Growing economic globalization and the Belt and Road Initiative have led to an increase in the cross-border population movements of Chinese people. With more than 60 million people in more than 160 countries and regions on all continents (Zheng et al., 2022), the Chinese diaspora is one of the world’s largest. However, a huge number of children remain in China when their parents move overseas. Recent studies have shown that these left-behind children experience poorer academic adjustment (Wang et al., 2021), more loneliness (Ling et al., 2017), and lower levels of peer support (Lan and Wang, 2019), fueling concern and motivating researchers to find ways to help them adjust socially and academically.

School is the primary setting for children’s learning and development, and children’s academic achievement and personal psychological growth are significantly influenced by their ability to adapt to environmental changes (Cleveland and Fisher, 2014). Additionally, as a crucial resource for future engagement in nation-building, low levels of school adjustment can easily result in false perceptions of oneself and one’s regional culture, especially for the left-behind children of diaspora whose discomfort with school can negatively affect their ability to socialize (Keshavarz et al., 2010). At the same time, COVID-19 has limited cross-country mobility and made it harder for parents and children to communicate offline, reducing the support that children need to cope with negative stimuli. With increasing numbers of children left behind by overseas parents, who they cannot see due to the impact of COVID-19, efforts to facilitate school adjustment are increasingly important.

### The relationship between parent–child attachment and school adjustment

*Parent–child attachment* refers to the lasting and stable emotional bond between parents and children, it can be divided into three types: secure attachment, avoidant attachment and contradictory attachment (Bowlby, 1977). It is the most important foundation of caregiver-child dyadic relationships within the family and has a significant impact on an individual’s interaction with peers. Attachment theory holds that children whose interactions with parents are characterized by care and trust develop a positive internal working model of social interaction. Otherwise, individuals tend to develop negative internal working models, lack the willingness to socialize and struggle to interact positively with others (Bowlby, 1982). Subsequent research has also confirmed that children with less secure levels of attachment tend to lack trust and communication with their parents and are more likely to engage in problematic behaviors, while children with higher parent–child secure attachment levels are more likely to develop prosocial behaviors (Laible, 2007).

*School adjustment* is the extent to which students can adapt to the demands, academic tasks, and interpersonal relationships of the school system, as well as feel comfortable, engaged, and accepted in the school environment (Ladd et al., 1997). It includes academic, behavioral, and emotional forms of adaptation. Numerous studies have shown that parent–child attachment is positively associated with children’s levels of school adjustment (Bardack et al., 2017). Among children with disabilities, parent–child attachment also positively affects children’s participation in school activities, academic achievement, and behavior (Huang et al., 2018). Essentially, a healthy family relationship is the basis on which a child goes on to develop a healthy personality (Ooi et al., 2006). Based on the findings summarized above, we proposed our first hypothesis.

*Hypothesis 1* (*H1*): Parent–child secure attachment is significantly and positively correlated with school adaptation among the left-behind children of the Chinese diaspora.

### The mediating role of peer relationships

Peers are significant individuals in children’s lives and an essential external resource in the process of individual socialization. *Peer relationships* are defined as interpersonal ties formed during interactions between people of a similar age and psychological development. Peers provide companionship, emotional support, and recreational opportunities as children grow and develop (Oberle et al., 2018). Peer relationships play a significant role in children’s acceptance, integration, and participation in the school environment. These links also influence psychological variables related to the environment, such as children’s attitudes to school, their drive to succeed, and their self-control. Conversely, negative peer relationships may cause youngsters to retreat from and avoid social situations, which can lead to psychological issues like social anxiety and introversion (Molden et al., 2009). Studies have confirmed that close peer relationships can affect children’s ability to regulate their conduct and develop new social attitudes (Heinze et al., 2018). Similarly, peer relationships can help children who have been left behind: the formation of emotional ties in peer relationships can help left-behind kids adjust cognitively, emotionally, and behaviorally by making up for the absence of immediate family members (Ling et al., 2017). Overall, it is likely that peer relationships are an important factor influencing the integration and adjustment of the left-behind children of the diaspora.

Early parent–child interactions can influence the development and direction of children’s future interpersonal relationships (Bernier et al., 2015). Effective parent–child attachment can help children develop positive self-perceptions and expectations of peer behavior, making individuals more likely to develop a sense of trust. Research has also shown that high-quality parent–child attachment helps children to develop better emotional regulation, which in turn enhances their sense of inclusion in peer interactions and their self-identity in social interactions (Healy and Sanders, 2018). Conversely, children with lower levels of parent–child secure attachment are more likely to exhibit adverse signs of physical and emotional development that negatively predict the quality of their peer interactions and social identity (Duffy and Nesdale, 2009). Given these findings, we proposed our second hypothesis.

*Hypothesis 2* (*H2*): Peer relationships mediate the relationship between parent–child attachment and school adaptation among the left-behind children of the Chinese diaspora.

### The mediating role of hometown identity

*Hometown identity* is the psychological process by which individuals construct meaning and seek belonging based on a specific local culture. Its formation is primarily influenced by the public order and customs, behavioral norms, and sense of belonging that individuals experience in their hometown society (Chen et al., 2020). Ecological systems theory views the physical and psychological environment of the family as a micro-system that gradually extends to the hometown and society. The family, hometown, and the social setting where students live and learn (school) are the projections of children’s psychological activities during this process. This is an internal structure of mutual influence, meaning that children’s acceptance of their home environment affects their psychological engagement in this environment (Bronfenbrenner, 1979). Furthermore, children’s adaptation to the home environment also affects their feelings in the social environment (school), which directly influences how they perceive and interact with the place and group to which they belong (Liu et al., 2022). In this sense, therefore, an individual’s local identity and identification with his or her home town inevitably affects the extent to which he or she adapts to the school environment in a self sense (Zhao et al., 2022). It has been shown that children’s hometown identity is positively associated with their level of school adjustment; for example, one study found that the degree to which ethnic identity was locally integrated predicted the school adjustment of migrant children (Spiegler et al., 2018).

Thus, the group affiliation and local identity of children left behind by overseas parents affect their ability to adjust to school. At the same time, the parent–child interaction process consists of a psychological process of seeking self, collective and territorial belonging (Seror, 2022). Children with strong parent–child secure attachments can meet their emotional developmental needs and generate many positive psychological experiences. These positive experiences insulate them from negative emotional shocks, enhance their identification with the regional cultural environment in which they grow up, and reduce the risk of confusion over issues related to belonging (Freeman et al., 2022). On the other hand, children whose parent–child secure attachments are weaker may struggle more to develop a social-cultural sense of regional belonging and may not develop adaptively in the emotional, cognitive, and behavioral domains. On this basis, we formulated our third hypothesis.

*Hypothesis 3* (*H3*): Hometown identity mediates the relationship between parent–child attachment and school adjustment among the left-behind children of the Chinese diaspora.

### The current study

Although left-behind children have been studied extensively, some gaps in knowledge remain unfilled. For instance, most earlier studies concentrated on the mental health issues of left-behind children while virtually ignoring their difficulties in adjusting to schooling, which may considerably impact their futures. In grades 4–8, left-behind children are at an important stage of moral development (Hu et al., 2014), so it is crucial to closely investigate their learning and adaptation. Additionally, past research has not adequately examined the individual circumstances of left-behind children. Numerous studies have concentrated on the impact of environmental factors (Ye and Pan, 2011), but we should pay more attention to themselves. Our overall aim was thus to investigate the elements influencing the school adjustment of left-behind children and the underlying mechanism of action.

## Materials and methods

### Participants

This study was conducted in October 2021 and April 2022, after the COVID-19 pandemic. We used whole-group sampling to select students at grades 3–8 from 5 schools in Wenzhou City, Zhejiang Province, eastern China. A total of 1,047 children participated in the survey. Many of Wenzhou’s former inhabitants have relocated overseas, making the population highly suited to our research aims. Based on Zhao et al.’s criteria (Zhao et al., 2012), 59 unsuitable responses were excluded, leaving a valid response rate of >90% (988 children). The sample included 379 children with both parents abroad (49.06% female; M = 12.44 years old, SD = 0.53), 132 children with fathers only overseas (48.78% female, M = 11.57 years, SD = 0.42), and 95 children whose mothers only were abroad (46.67% female, M = 12.09 years, SD = 0.57). There were 382 children whose parents had not gone overseas (52.64% female, M = 11.31 years old, SD = 0.43). Thus, among the 606 children of one or two overseas parents, those born abroad accounted for the largest proportion, at 49.06%, while those born in their hometown made up 39.62% of the sample. Overall, 32.16% of the children had been left behind for 1–2 years; 39.45% for 3–5 years; and 28.39% for more than 5 years.

### Measures

#### Parent–child attachment questionnaire

The Parent–child Attachment Questionnaire developed by Armsden and Greenberg (1987) and revised by Zhao et al. (2019). It contains two sub-sections covering father-child and mother–child secure attachment. Each subsection consists of 10 items divided into three dimensions: trust, communication, and detachment, and is scored on a 5-point scale (1 = never, 5 = always). Following scholarly precedent, the father-child and mother–child attachment scores were combined to obtain the total parent–child attachment score (Di Folco et al., 2017). This was the sum of the scores from the trust and communication dimensions minus the score from the detachment dimension. The internal consistency coefficient of the parent–child attachment questionnaire was 0.897.

#### Student peer relationship scale

Students’ relationships with their peers were evaluated using the Student Peer Relationship Scale created by Rose and Asher (1999). The 16-item survey is graded on a 5-point scale (1 = “not at all,” 5 = “totally”). The scale is divided into three dimensions: “welcome,” “exclusion,” and “isolation.” The higher the total score on the peer relationship scale, the better the peer relationship. The scale’s internal consistency coefficient for this study was 0.912.

#### Hometown identity scale

The Hometown Identity Scale developed by Hwang was used to evaluate students’ hometown identity status (Hwang and Chang, 2011). The questionnaire consists of 18 items and is scored on a 5-point scale (1 = “not at all,” 5 = “completely”). In this study, the internal consistency coefficient of the Hometown Identity Scale was 0.923.

#### School adaptation scale

School Adaptation was assessed using a 13-item Chinese version of the original scale developed by Zhang et al. (2012). A 5-point Likert-type scale was used to score responses (1 = “not at all,” 5 = “fully”). Five items assessed academic adjustment (e.g., “I can connect old and new knowledge to learn”), four measured interpersonal adjustment (e.g., “I feel that others treat me in a friendly way”), and four measured emotional adjustment (e.g., “I have a way to make myself happy when things go wrong”). The mean score of the 13 items was taken, with higher scores indicating greater school adjustment. In the current study, the internal consistency coefficient of this scale was 0.889.

### Research procedures

We sought the help of principals and teachers to access the study participants. Then, we contacted the participants’ parents or guardians online and obtained their permission. Before data collection, all questionnaires were administered using group tests with the assistance of classroom teachers, and the students were assured of the voluntary and confidential nature of this research. Students independently completed the four scales during classroom hours or immediately after school. After the questionnaires were returned, common method bias tests and correlation analyses were conducted using SPSS 26.0, and mediated effects analyses were conducted using PROCESS for SPSS developed by Hayes (2013).

## Results

### Common method deviation test

The Harman one-way test was used to test for common method bias. The results showed that there were 16 factors with eigenvalues greater than 1 and the variance explained by the first factor was 27.173%, which was less than the critical criterion of 40%, indicating that the possibility of common method bias in this study was low.

### Correlation analysis of each latent variable

The results of the correlation analysis of the variables of parent–child secure attachment, peer relationship, school adjustment, and hometown identity (see Table 1) show that the four variables were significantly correlated with each other. Significant correlations were detected between the following: peer relationships and parent–child attachment (*r* = 0.510, *p* < 0.01), peer relationships and school adjustment (*r* = 0.617, *p* < 0.01), hometown identity and school adjustment (*r* = 0.504, *p* < 0.01), school adjustment and parent–child attachment (*r* = 0.496, *p* < 0.01), hometown identity and parent–child attachment (*r* = 0.375, *p* < 0.01), and peer relationships and hometown identity (*r* = 0.430, *p* < 0.01).

**Table 1:** Correlation analysis among variables for left-behind children of the diaspora (*n* = 606).

	1	2	3	4
1. Parent–child attachment	1			
2. Peer relationships	0.510**	1		
3. School adaptation	0.496**	0.617**	1	
4. Hometown identity	0.375**	0.430**	0.504**	1

**p < 0.01.

### The test of the chain mediating role of peer relations and hometown identity

The results showed a two-by-two positive correlation between the variables and met the statistical requirements for further tests of their mediating effects of peer relationships and hometown identity. A chained mediating effects test was conducted using Model 6 in the SPSS macro program, with gender and grade as control variables, parent–child attachment as the independent variable, peer relationship and hometown identity as mediating variables, and school adjustment as the dependent variable for analysis.

The results of the regression analysis (Table 2) showed that parent–child attachment significantly and positively predicted school adjustment (*β* = 0.467, *p* < 0. 001). After peer relationships and hometown identity were included in the regression equation, parent–child attachment significantly and positively predicted peer relationships (*β* = 0.503, *p* < 0.001) and hometown identity (*β* = 0.226, *p* < 0.01). Parent–child attachment remained a significant positive predictor of school adjustment (*β* = 0.310, *p* < 0.001) after including peer relationships (*β* = 0.503, *p* < 0.001) and hometown identity (*β* = 0.226, *p* < 0.01) while peer relationships were a significant positive predictor of home town identity (*β* = 0.314, *p* < 0.001) and school adjustment (*β* = 0.346, *p* < 0.001).

**Table 2:** Analysis of the regression relationship between the variables.

Regression equation (N = 606)		Overall fit coefficient			Significance of regression coefficients	
Resulting variables	Predictor variables	R	R^2^	F	β	t
School adaptation		0.650	0.423	50.357		
	Gender				−0.026	−1.649
	Grade Level				0.054	1.586
	Parent–child attachment				0.467	7.311***
Companionship		0.515	0.265	24.744***		
	Gender				−0.031	−0.512
	Grade				−0.043	−0.691
	Parent–child attachment				0.503	8.262***
Hometown identity		0.475	0.225	14.907***		
	Gender				0.052	0.838
	Grade Level				−0.016	−0.259
	Parent–child attachment				0.226	3.118**
	Companionship				0.314	4.381***
School adaptation		0.732	0.536	47.118***		
	Gender				−0.023	−0.479
	Grade				−0.102	−2.063*
	Parent–child attachment				0.310	5.389***
	Companionship				0.346	5.948***
	Hometown identity				0.228	4.203***

*p < 0.05; **p < 0.01; ***p < 0.001. All continuous variables in the model were standardized; gender and grade were dummy coded, 1 = male, 2 = female.

**Figure 1:**
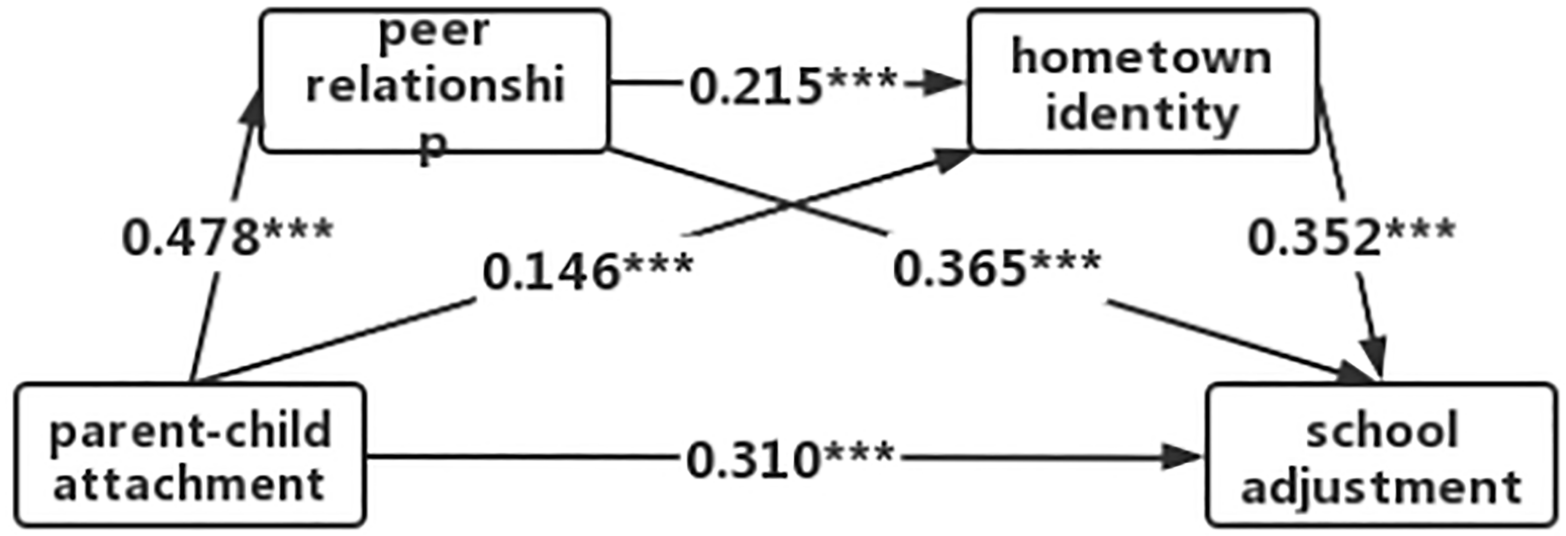
Mediated effects pathway test. ***p < 0.001.

The results of the quantitative analysis of the mediating effects (see Figure 1; Table 3) show that peer relationships and hometown identity played a significant mediating role in the link between parent–child attachment and school adjustment among children whose overseas parents had left them behind, with a total standardized mediating effect value of 0.262, accounting for 51.35% of the total effect of parent–child attachment on school adjustment (effect value of 0.310). The mediating effect consisted of three profile effects: indirect effect 1 (with an effect size of 0.174) for the parent–child attachment → peer relationship → school adjustment pathway; indirect effect 2 (effect size: 0.051) for the parent–child attachment → hometown identity → school adjustment pathway; and indirect effect 3 (0.036) for the parent–child attachment → peer relationship → hometown identity → school adjustment pathway. These three indirect effects accounted for 29.68, 12.18, and 14.31% of the total effect, respectively. The 95% confidence intervals for all three indirect effects did not contain 0, indicating that all three were significant.

**Table 3:** Analysis of intermediary effect sizes (N = 606).

Effect	Indirect effect value	Boot SE	Boot LLCI	Boot ULCI	Relative intermediary effects
Total indirect effect	0.262	0.038	0.189	0.341	51.35%
Indirect effect 1	0.174	0.036	0.104	0.247	29.68%
Indirect effect 2	0.051	0.020	0.017	0.098	12.18%
Indirect effect 3	0.036	0.013	0.016	0.065	14.31%
Compare 1	0.123	0.046	0.031	0.210	
Compare 2	0.138	0.041	0.055	0.217	
Compare 3	0.015	0.022	−0.028	0.060	

Boot SE, Boot LLCI, and Boot ULCI refer to the standard errors, lower and upper 95% confidence intervals of the indirect effects estimated by the bias-corrected percentile Bootstrap method, respectively; Indirect effect 1: Parent–child attachment → peer relationship → school adjustment; Indirect effect 2: Parent–child attachment → hometown identity → school adjustment; Indirect effect 3: Parent–child attachment → peer relationships → hometown identity → school adjustment.

Next, a two-by-two comparison of indirect effects across pathways was conducted to examine whether there were significant pathway differences: Comparison 1 shows that the bootstrap at a 95% confidence interval for the difference between indirect effects 1 and 2 did not contain 0, indicating that these two effects differed significantly, with indirect effect 1 greater than indirect effect 2. Similarly, indirect effect 1 was greater than indirect effect 3, while the difference between indirect effects 1 and 2 was greater than indirect effect 2.

## Discussion

### The positive predictive effect of parent–child attachment on the school adjustment of left-behind children

The findings indicated that parent–child attachment was significantly and positively related to school adjustment among children left behind by one or both diasporic parents, with structural equation modeling confirming parent–child attachment as a significant and positive predictor of school adjustment. Notably, the pathways modeled in this study show that peer relationships and hometown identity mediated the effect of parent–child attachment on the school adjustment of left-behind children, thereby supporting the ecological system theory that parent–child attachment will directly influence the ability of left-behind children to adjust to school as well as indirectly influencing it through peer relationships and hometown identity. In addition, the relationship between parent–child attachment and school adjustment was even more pronounced among children left behind by overseas parents, where the isolation of children from their parents—the “defining characteristic” of such children—is the core indicator that distinguishes children left behind from those not left behind (Chung et al., 2021). Children separated from their parents for sustained periods demonstrate significantly lower levels of parent–child attachment than those who remain together (Zhang and Deng, 2022), and relatively lower levels of school adjustment, pointing to the developmental risks to left-behind children (Doumen et al., 2012). Interventions might therefore center on improving parent–child attachment as a means of enhancing left-behind children’s adjustment to their schools, such as improving the quality of parent–child communication (Zhang et al., 2021).

### The mediating effects of peer relations and hometown identity

The findings show that parent–child attachment may influence the school adjustment of children left behind by overseas parents through the mediating role of peer relationships or hometown identity. Furthermore, specific pathway analysis revealed that parent–child attachment was a more powerful positive predictor of peer relationships than of hometown identity, while peer relationships positively predicted school adjustment more strongly than hometown identity. Attachment theory suggests that securely attached individuals tend to possess positive self-other schemata and that strong parent–child attachment promotes positive cognitive, emotional, and behavioral development. On the one hand, individuals with high-quality parent–child attachments are more confident and trustworthy (Gómez-Ortiz et al., 2020). They are more likely to develop high-quality friendships with peers in their daily school lives. On the other hand, individuals with high-quality parent–child attachments are more likely to perceive care and support from their peers and adjust to the school environment accordingly (Ratelle et al., 2017). Thus, parent–child attachment is more closely related to peer relationships. At the same time, good peer relationships not only enable children to acquire social values and develop and exercise their social skills but also contribute to healthy cognitive and emotional development (Rose et al., 2022). Conversely, poor peer relationships may obstruct children’s ability to make the necessary school adjustments in the academic, emotional, and behavioral domains. Ladd’s Interpersonal Model of School Adjustment emphasizes the role of children’s peer relationships (including peer attachments) in the process of adapting to educational environments (Birch and Ladd, 1997). Peer attachment is both positively correlated with and predicts school adjustment (Laible et al., 2000), and is thus more closely related to school adjustment than hometown identity.

### The chain mediating effects of peer relations and hometown identity

Good peer relationships provide the necessary emotional support for children to grow as individuals, reducing the risk of them developing psychological problems such as depression and anxiety (Watson, 2019). According to the Interpersonal Model of School Adjustment, interpersonal relationships that people form in groups at a young age can help individuals adjust to complex social situations and directly impact their understanding (Ladd, 1988). This study views hometown identity as both a psychological notion that creates a person’s self-image and a method that people use to process, use, and modify local information about themselves (Romera Félix et al., 2022). In this way, peer relationships are an important influence on the way individuals construct their self-concept and sense of belonging to a particular vernacular culture. The evidence from the present study also validated, for the first time, the proposition that peer relationships and hometown identity function as a chain mediator in parent–child attachment and school adjustment, and to be more specific, that the chain partially mediating the relationship between parent–child relationship and school adjustment. One study showed that weaker hometown identity among Chinese immigrants was significantly associated with poorer interpersonal relationships, a relationship moderated by the degree of social adjustment (Lin et al., 2022).

Besides, we found that there is a transmission pathway of parent–child attachment-peer relationship-hometown identity-school adjustment. This implies that when parent–child attachment can be maintained at a certain level among left-behind children of overseas Chinese, it may enable them to better merge into the community (Xu et al., 2015), establish healthier companionship with peers (Li et al., 2011), access better local identity and a sense of hometown belonging, which leads to a better adaptive psychological performance to their school (Mattiace and de Mola, 2015); therefore, a seamless connection between parent–child attachment, peer relationships, hometown identity, and school adjustment is crucial for this as yet unclear research field (Zou et al., 2021). As it is capable of supporting the left-behind children of overseas Chinese to establish a stable mental model to handle the adverse effects of spatial separation with their parents and prompting their environmental adaptability, so that the conversion effectiveness of parent–child relationship on individuals’ school environment perception, adjustment and adaptability will be further optimized.

## Conclusion

In summary, the main findings of this study are as follows: (1) Parent–child attachment was positively correlated with school adjustment among left-behind children of the Chinese diaspora; (2) Among these children, peer relationships and hometown identity partially mediated the relationship between parent–child attachment and school adjustment; (3) Peer relationships and hometown identity played a chain mediating role in the relationship between parent–child attachment and school adjustment.

## Limitations

Without underplaying the contribution of the present study, several limitations must also be mentioned. Firstly, because the study population was confined to randomly selected students from one specific city (Wenzhou, Zhejiang Province), it is not genuinely representative. Future research should therefore increase the sample size to fully represent the target population. In addition, this was a cross-sectional study and thus cannot accurately determine the causal relationship between the variables. Therefore, future studies should take a longitudinal approach to address this deficiency.

## Data availability statement

The raw data supporting the conclusions of this article will be made available by the authors, without undue reservation.

## Ethics statement

The studies involving human participants were reviewed and approved by Institutional Review Board of Wenzhou University (protocol code 2017). Written informed consent to participate in this study was provided by the participants’ legal guardian/next of kin.

## Author contributions

XY and HZ: conceptualization. HZ and ZL: methodology, data curation, and writing-original draft preparation. HZ: formal analysis. XY: investigation. XY and CD: writing-review and editing, supervision, and funding. All authors contributed to the article and approved the submitted version.

## Funding

This study received funding from the following sources: “Research on the Construction of the Evaluation System for Practical Education in Colleges and Universities from the Perspective of Education Evaluation Reform in the New Era,” China’s 2022 Ministry of Education Humanities and Social Sciences Research Special Task Project (China), Grant No.22JDSZ3169; “Research on the Formation and Cultivation Mechanism of the Family Sentiment of Overseas Chinese from the Perspective of Embodiment,” Zhejiang Provincial Social Science Planning Major Project (China), Grant No. 22JCXK02ZD.

## Conflict of interest

The authors declare that the research was conducted in the absence of any commercial or financial relationships that could be construed as a potential conflict of interest.

## Publisher’s note

All claims expressed in this article are solely those of the authors and do not necessarily represent those of their affiliated organizations, or those of the publisher, the editors and the reviewers. Any product that may be evaluated in this article, or claim that may be made by its manufacturer, is not guaranteed or endorsed by the publisher.

## References

[ref1] Armsden G. C. Greenberg M. T. (1987). The inventory of parent and peer attachment. Individual differences and their relationship to psychological well-being. J. Youth Adolesc. 16, 427–454. doi: 10.1007/BF02202939, PMID: 24277469

[ref2] Bardack S. Herbers J. E. Obradović J. (2017). Unique contributions of dynamic versus global measures of parent-child interaction quality in predicting school adjustment. J. Fam. Psychol. 31, 649–658. doi: 10.1037/fam0000296, PMID: 28277709

[ref3] Bernier A. Beauchamp M. H. Carlson S. M. Lalonde G. (2015). A secure base from which to regulate: attachment security in toddlerhood as a predictor of executive functioning at school entry. Dev. Psychol. 51, 1177–1189. doi: 10.1037/dev0000032, PMID: 26192039

[ref4] Birch S. H. Ladd G. W. (1997). The teacher-child relationship and children’s early school adjustment. J. Sch. Psychol. 35, 61–79. doi: 10.1016/S0022-4405(96)00029-5

[ref5] Bowlby J. (1977). The making and breaking of affectional bonds: I Aetiology and psychopathology in the light of attachment theory. Brit J Psychiatry. 130, 201–210. doi: 10.1192/bjp.130.3.201, PMID: 843768

[ref6] Bowlby J. (1982). Attachment and loss: retrospect and prospect. Am. J. Orthop. 52, 664–678. doi: 10.1111/j.1939-0025.1982.tb01456.x, PMID: 7148988

[ref7] Bronfenbrenner U. (1979). The ecology of human development: Experiments by nature and design. Cambridge, MA: Harvard University Press.

[ref8] Chen H. Zhu Z. Chang J. Gao Y. (2020). The effects of social integration and hometown identity on the life satisfaction of Chinese rural migrants: the mediating and moderating effects of a sense of belonging in the host city. Health Qual. Life Outcomes 18, 1–9. doi: 10.1186/s12955-020-01415-y 32505205 PMC7275305

[ref9] Chung S. Zhou Q. Kho C. Main A. (2021). Parent-child conflict profiles in Chinese American immigrant families: links to sociocultural factors and school-age children’s psychological adjustment. Fam. Process 60, 169–185. doi: 10.1111/famp.12546, PMID: 32432357 PMC7677213

[ref10] Cleveland B. Fisher K. (2014). The evaluation of physical learning environments: a critical review of the literature. Learn. Environ. Res. 17, 1–28. doi: 10.1007/s10984-013-9149-3

[ref11] Di Folco S. Messina S. Zavattini G. C.. (2017). Attachment to mother and father at transition to middle childhood. J. Child Fam. Stud. 26, 721–733. doi: 10.1007/s10826-016-0602-7, PMID: 28239249 PMC5306151

[ref12] Doumen S. Smits I. Luyckx K. Duriez B. Vanhalst J. Verschueren K.. (2012). Identity and perceived peer relationship quality in emerging adulthood: the mediating role of attachment-related emotions. J. Adolesc. 35, 1417–1425. doi: 10.1016/j.adolescence.2012.01.003, PMID: 22325118

[ref13] Duffy A. L. Nesdale D. (2009). Peer groups, social identity, and children’s bullying behavior. Soc. Dev. 18, 121–139. doi: 10.1111/j.1467-9507.2008.00484.x

[ref14] Freeman C. Latai N. A. Schaaf M.. (2022). Identity, belonging and place attachment amongst Pacific Island children: a photographic analysis. Child Geog. 1–16. doi: 10.1080/14733285.2022.2039900

[ref15] Gómez-Ortiz O. Zea R. Ortega-Ruiz R. Romera E. M. (2020). Perception and social motivation: predictive elements of social anxiety and adjustment in adolescents. Psicologia Educativa 26, 49–55. doi: 10.5093/psed2019a11

[ref16] Hayes A. F. (2013). Introduction to mediation, moderation, and conditional process analysis: A regression-based approach. New York: Guilford Press.

[ref17] Healy K. L. Sanders M. R. (2018). Mechanisms through which supportive relationships with parents and peers mitigate victimization, depression and internalizing problems in children bullied by peers. Child Psychiatry Hum. Dev. 49, 800–813. doi: 10.1007/s10578-018-0793-9, PMID: 29473091

[ref18] Heinze J. E. Cook S. H. Wood E. P. (2018). Friendship attachment style moderates the effect of adolescent exposure to violence on emerging adult depression and anxiety trajectories. J. Youth Adolesc. 47, 177–193. doi: 10.1007/s10964-017-0729-x, PMID: 28815358 PMC5750100

[ref19] Hu H. Lu S. Huang C. C. (2014). The psychological and behavioral outcomes of migrant and left-behind children in China. Child Youth Serv. Rev. 46, 1–10. doi: 10.1016/j.childyouth.2014.07.021

[ref20] Huang C. Y. Ching-Yun Y. Wu I. (2018). Relationships between the parent-child interaction, self-concept, and school adjustment of junior high school students with disabilities. J Res Educ Sci. 63:103. doi: 10.6209/JORIES.2018.63(1).04

[ref21] Hwang G. J. Chang H. F. (2011). A formative assessment-based mobile learning approach to improving the learning attitudes and achievements of students. Comput. Educ. 56, 1023–1031. doi: 10.1016/j.compedu.2010.12.002

[ref22] Keshavarz N. Nutbeam D. Rowling L. Khavarpour F. (2010). Schools as social complex adaptive systems: a new way to understand the challenges of introducing the health promoting schools concept. Soc. Sci. Med. 70, 1467–1474. doi: 10.1016/j.socscimed.2010.01.034, PMID: 20207059

[ref23] Ladd G. W. (1988). Friendship patterns and peer status during early and middle childhood. J. Dev. Behav. Pediatr. 9, 229–238. doi: 10.1097/00004703-198808000-00010 3063725

[ref24] Ladd G. Kochenderfer B. J. Coleman C. C. (1997). Classroom peer acceptance, friendship, and victimization: Destinct relation systems that contribute uniquely to Children’s school adjustment. Child Dev. 68, 1181–1197. doi: 10.1111/j.1467-8624.1997.tb01993.x, PMID: 9418233

[ref25] Laible D. (2007). Attachment with parents and peers in late adolescence: links with emotional competence and social behavior. Personal. Individ. Differ. 43, 1185–1197. doi: 10.1016/j.paid.2007.03.010

[ref26] Laible D. J. Carlo G. Raffaelli M. (2000). The differential relations of parent and peer attachment to adolescent adjustment. J. Youth Adolesc. 29, 45–59. doi: 10.1023/A:1005169004882

[ref27] Lan X. Wang W. (2019). Direct and interactive effects of peer support and resilience on psychosocial adjustment in emerging adults with early left-behind experiences. Psychol. Res. Behav. Manag. 12, 277–288. doi: 10.2147/PRBM.S202774, PMID: 31114409 PMC6489595

[ref28] Li Y. Doyle L. A. Kalvin C.. (2011). Peer relationships as a context for the development of school engagement during early adolescence. Int. J. Behav. Dev. 35, 329–342. doi: 10.1177/0165025411402578

[ref29] Lin S. Wu F. Liang Q. Li Z. Guo Y. (2022). From hometown to the host city? Migrants’ identity transition in urban China. Cities 122:103567. doi: 10.1016/j.cities.2022.103567

[ref30] Ling H. Fu E. Zhang J. R. (2017). Peer relationships of left-behind children in China moderate their loneliness. Soc. Behav. Personal. Int. J. 45, 901–913. doi: 10.2224/sbp.6021

[ref31] Liu Y. B. Hou X. Y. Chen B. B. (2022). Links between Chinese vocational school students’ perception of parents’ emotional support and school cooperation climate and their academic performance: the mediating role of school belonging. Front. Psychol. 13:952001. doi: 10.3389/fpsyg.2022.952001 35967675 PMC9374126

[ref32] Mattiace S. L. de Mola P. F. L. (2015). Yucatec Maya organizations in San Francisco, California: ethnic identity formation across migrant generations. Lat. Am. Res. Rev. 50, 201–215. doi: 10.1353/lar.2015.0019

[ref33] Molden D. C. Lucas G. M. Gardner W. L. Dean K. Knowles M. L. (2009). Motivations for prevention or promotion following social exclusion: being rejected versus being ignored. J. Pers. Soc. Psychol. 96, 415–431. doi: 10.1037/a0012958 19159140

[ref34] Oberle E. Guhn M. Gadermann A. M. Thomson K. Schonert-Reichl K. A. (2018). Positive mental health and supportive school environments: a population-level longitudinal study of dispositional optimism and school relationships in early adolescence. Soc. Sci. Med. 214, 154–161. doi: 10.1016/j.socscimed.2018.06.041, PMID: 30072159

[ref35] Ooi Y. P. Ang R. P. Fung D. S. S. Wong G. Cai Y. (2006). The impact of parent–child attachment on aggression, social stress and self-esteem. Sch. Psychol. Int. 27, 552–566. doi: 10.1177/0143034306073402

[ref36] Ratelle C. F. Duchesne S. Guay F. (2017). Predicting school adjustment from multiple perspectives on parental behaviors. J. Adolesc. 54, 60–72. doi: 10.1016/j.adolescence.2016.11.008, PMID: 27871016

[ref37] Romera Félix E. M. Luque González R. Ortega Ruiz R. Gómez Ortiz O. Camacho López A. (2022). Positive peer perception, social anxiety and classroom social adjustment as risk factors in peer victimization: a multilevel study. Psicothema 34, 110–116. doi: 10.7334/psicothema2021.37, PMID: 35048902

[ref38] Rose A. J. Asher S. R. (1999). Children’s goals and strategies in response to conflicts within a friendship. Dev. Psychol. 35, 69–79. doi: 10.1037/0012-1649.35.1.69, PMID: 9923465

[ref39] Rose A. J. Smith R. L. Schwartz-Mette R. A. Glick G. C. (2022). Friends’ discussions of interpersonal and noninterpersonal problems during early and middle adolescence: associations with co-rumination. Dev. Psychol. 445. doi: 10.1037/dev0001445, PMID: 36048101 PMC9758691

[ref40] Seror A. (2022). Child development in parent-child interactions. J. Polit. Econ. 130, 2462–2499. doi: 10.1086/720398

[ref41] Spiegler O. Sonnenberg K. Fassbender I. Kohl K. Leyendecker B. (2018). Ethnic and national identity development and school adjustment: a longitudinal study with Turkish immigrant-origin children. J. Cross-Cult. Psychol. 49, 1009–1026. doi: 10.1177/0022022118769773

[ref42] Su S. Li X. Lin D. Xu X. Zhu M. (2013). Psychological adjustment among left-behind children in rural China: the role of parental migration and parent–child communication. Child Care Health Dev. 39, 162–170. doi: 10.1111/j.1365-2214.2012.01400.x, PMID: 22708901

[ref43] Wang Y. Liu W. Wang W. Lin S. Lin D. Wang H. (2021). Left-behind children’s social adjustment and relationship with parental coping with children’s negative emotions during the COVID-19 pandemic in China. Int. J. Psychol. 56, 512–521. doi: 10.1002/ijop.12754, PMID: 33739446 PMC8251002

[ref44] Watson E. (2019). The mechanisms underpinning peer support: a literature review. J. Ment. Health 28, 677–688. doi: 10.1080/09638237.2017.1417559, PMID: 29260930

[ref45] Xu M. de Bakker M. Strijker D. Wu H. (2015). Effects of distance from home to campus on undergraduate place attachment and university experience in China. J. Environ. Psychol. 43, 95–104. doi: 10.1016/j.jenvp.2015.05.013

[ref46] Ye J. Pan L. (2011). Differentiated childhoods: impacts of rural labor migration on left-behind children in China. J. Peasant Stud. 38, 355–377. doi: 10.1080/03066150.2011.559012 21744548

[ref47] Zhang H. Deng C. (2022). The impact of parent-child attachment on school adjustment in left-behind children due to transnational parenting: the mediating role of peer relationships. Int. J. Environ. Res. Public Health 19, 69–89. doi: 10.3390/ijerph19126989 PMC9222539 35742238

[ref48] Zhang D. J. Gong L. Wang J. L. (2012). The revision of adolescent’s psychological Suzhi questionnaire. Int. J. Psychol. 47:19.

[ref49] Zhang Q. Shek D. T. L. Pan Y. (2021). Parent-child discrepancies in perceived parent-child communication and depressive symptoms in early adolescents in China. Int. J. Environ. Res. Public Health 18:12041. doi: 10.3390/ijerph182212041, PMID: 34831792 PMC8624406

[ref50] Zhao S. Chen X. Li D. Liu J. Yang P. (2022). Maternal encouragement of competitiveness and school adjustment in Chinese adolescents. J. Fam. Psychol. 1034. doi: 10.1037/fam0001034, PMID: 36136829

[ref51] Zhao J. Gao F. Xu Y. Sun Y. Han L. (2019). The relationship between shyness and aggression: the multiple mediation of peer victimization and security and the moderation of parent-child attachment. Pers. Individ. Differ. 156:109733. doi: 10.1016/j.paid.2019.109733

[ref52] Zhao J. X. Xing X. P. Wang M. F. (2012). Psychometric properties of the Spence children’s anxiety scale (SCAS) in mainland Chinese children and adolescents. J. Anxiety Disord. 26, 728–736. doi: 10.1016/j.janxdis.2012.05.006, PMID: 22858899

[ref53] Zheng X. Fang Z. Wang Y. Fang X. (2022). When left-behind children become adults and parents: the long-term human capital consequences of parental absence in China. China Econ. Rev. 101821. doi: 10.1016/j.chieco.2022.101821

[ref54] Zou Y. Meng F. Li Q. (2021). Chinese diaspora tourists’ emotional experiences and ancestral hometown attachment. Tour. Manag. Perspect. 37:100768. doi: 10.1016/j.tmp.2020.100768

